# Controlling bacteriological contamination of environmental surfaces at the biological isolation and containment unit of a veterinary teaching hospital

**DOI:** 10.1186/s13620-021-00197-z

**Published:** 2021-06-28

**Authors:** C. Verdial, C. Carneiro, I. Machado, L. Tavares, V. Almeida, M. Oliveira, S. Gil

**Affiliations:** 1 Faculty of Veterinary Medicine, University of Lisbon, Av. Universidade Técnica, 1300-477 Lisbon, Portugal; 2 CIISA - Centre for Interdisciplinary Research in Animal Health, Faculty of Veterinary Medicine, University of Lisbon, Av. Universidade Técnica, 1300-477 Lisbon, Portugal; 3 Veterinary Teaching Hospital, Faculty of Veterinary Medicine, University of Lisbon, Av. Universidade Técnica, 1300-477 Lisbon, Portugal

**Keywords:** Nosocomial infection, Surveillance, Bacteriological control, Hospital environment

## Abstract

**Background:**

The Biological Isolation and Containment Unit (BICU) is a subunit of the Teaching Hospital of the Faculty of Veterinary Medicine of the University of Lisbon, Portugal, for the admission of animals with confirmed infectious diseases or under clinical suspicion and waiting for a diagnosis. As a high-risk environment for the transmission of infectious agents, it is extremely important to implement programs for the surveillance of nosocomial microorganisms in these facilities. The purpose of this study was to evaluate the level of bacterial contamination of the BICU environmental surfaces and to implement corrective actions on disinfection protocols. Swab samples were collected from selected environmental surfaces in 3 different areas of the BICU (isolation, work, and preparatory rooms) to evaluate the total aerobic bacterial load and investigate the presence of 4 nosocomial microorganisms: vancomycin-resistant *Enterococcus* spp., methicillin-resistant *Staphylococcus aureus*, 3rd-generation cephalosporin-resistant *Escherichia coli*, and carbapenem-resistant *Pseudomonas aeruginosa*. Bacterial quantification was performed by using non-selective media, while specific selective media were used for the isolation of the target microorganisms. Isolates were identified based on their macro and microscopic characteristics and their biochemical profile. Subsequently, new disinfection protocols were implemented, and their effectiveness evaluated.

**Results:**

The surfaces with the highest bacterial load in the isolation, preparatory, and worker’s rooms were the cages, hand-held sponge, and telephone, respectively. Regarding the 4 pathogens investigated, *Enterococcus* spp. were the most frequently isolated (11.3%), followed by *E. coli* (1.5%) and *P. aeruginosa* (1.5%). One of the *P. aeruginosa* isolates obtained was resistant to imipenem. In the end, new disinfection protocols were implemented, which proved to be effective in reducing bacterial counts by 99.99% in cages and the sponge, and by 90 to 99% on the telephone.

**Conclusions:**

This study allows to conclude that the cages and the human contact surfaces were the most contaminated in the isolation rooms. Nevertheless, the new disinfection strategies seemed to be effective in reducing environmental contamination, including by some potentially nosocomial agents, although more samples must be analyzed for definitive conclusions. These results may contribute to highlight the importance of infection prevention and control measures, as fundamental tools to reduce the spread of infectious agents in the hospital environment.

## Background

Each year, nosocomial infections (NI) are responsible for thousands of human deaths worldwide and have an estimated economic burden of billions of dollars in the USA alone [1]. These infections are acquired during hospitalization and have been associated with prolonged hospital stays, invasive medical procedures (e.g. surgeries, urinary catheterization, intubation), previous antibiotic treatments, and immunosuppression [2]. For those reasons, they are an inherent risk in medical practice, being currently a major concern worldwide mainly due to the frequent involvement of multidrug-resistant (MDR) bacteria [3].

In veterinary medicine, there are limited data concerning this problem, but reports of NI outbreaks in veterinary facilities are increasing [4–7], with one study revealing that 82% of the participating hospitals reported at least one NI outbreak in the previous 5 years [4].

These infections can be acquired from endogenous sources, being caused by opportunistic microorganisms already present in the individual himself, or from exogenous sources, being disseminated by other individuals or even the hospital environment [8]. The role played by hospital contaminated surfaces was once thought to be negligible in the transmission of such infections [9]; however, nowadays it is known that environmental surfaces are a reservoir of several microorganisms that can easily contaminate the hands and equipment of healthcare workers, which, in turn, can work as vehicles for the transmission of these pathogens to patients [10, 11]. Thereby, the implementation of an active surveillance system in healthcare facilities is highly recommended [12]. With the appropriate control measures, it is possible to prevent (or minimize) the direct exposure of healthcare workers to multiple biological hazards, as well as the indirect exposure of patients and even clients through contact with contaminated medical equipment or hospital surfaces [8]. In this context, isolation units are crucial to allow the immediate transference of high-risk infectious patients to these high-security facilities, thus limiting the circulation of potentially infectious agents in other hospital areas [8, 13].

Little is known about microbiological control in isolation units, especially in veterinary hospitals. However, several authors have demonstrated that hospital contamination with nosocomial agents is also a main concern in veterinary facilities [14–19]. Considering that the Biological Isolation and Containment Unit (BICU) is responsible for the hospitalization of patients colonized or infected with MDR bacteria, the main priorities of this study were to identify the environmental surfaces with the highest bacterial load, to recognize the presence of potential nosocomial agents and to implement appropriate corrective actions on current disinfection protocols.

## Results

### Bacterial identification-

From the 204 environmental samples tested, it was possible to obtain 29 isolates presumptively identified as belonging to 3 of the 4 target bacterial species (Table 1). It was not possible to isolate *Staphylococcus aureus* from any of the sampled surfaces.

**Table 1:** Distribution of isolated bacteria by the BICU sampled surfaces

Surface sampled	***Enterococcus*** spp.	***E. coli***	***P. aeruginosa***
Doorknob (*n* = 24)	2 (8.3%)	0	0
Faucets (*n* = 24)	3 (12.5%)	0	1 (4.2%)
Cabinets (*n* = 24)	3 (12.5%)	0	0
Cabinet handles 3^a^ (*n* = 24)	5 (20.8%)	0	0
Cabinet handles 2^b^ (*n* = 24)	4 (16.6%)	0	0
Examination tables (*n* = 24)	1 (4.2%)	0	0
Cages (*n* = 24)	4 (16.6%)	2 (8.3%)	0
Telephone (*n* = 6)	0	0	0
Keyboard (*n* = 6)	0	0	0
Mouse (*n* = 6)	0	0	0
Bowls (*n* = 6)	0	0	1 (16.6%)
Countertop (*n* = 6)	0	0	0
Hand-held sponge (*n* = 6)	1 (16.6%)	1 (16.6%)	1 (16.6%)
**TOTAL (*n*** **= 204)**	**23 (11.3%)**	**3 (1.5%)**	**3 (1.5%)**

^a^ Pool of the first 3 cabinet handles

^b^ Pool of the last 2 cabinet handles

*Enterococcus* spp. were isolated from approximately 11% (23/204) of the samples, having been recovered from all surfaces in the isolation rooms (Table 1); however, no relation was found between the presence of these bacteria and the different sampled surfaces (*p* = 0.963). All of these isolates (23/23) were susceptible to vancomycin, according to the Clinical and Laboratory Standards Institute (CLSI) guidelines [20].

*Escherichia coli* were identified in approximately 1.5% (3/204) of the samples. These isolates were recovered from the cages, the hand-held sponge, and the food/water bowls, although without associated statistical significance (*p* = 0.131). According to the susceptibility ranges established by the CLSI [20], all these isolates (3/3) were susceptible to the tested antibiotics and proven to be non ESBL-producing bacteria.

It was possible to isolate *Pseudomonas aeruginosa* from approximately 1.5% (3/204) of the samples, including from the faucets, the hand-held sponge, and the food/water bowls (Table 1). Once again, no statistically significant association was found between the presence of these bacterial species and the sampled surfaces (*p* = 0.081). Isolates susceptibility to imipenem was evaluated and, according to the ranges established by the CLSI [20], two were shown to be susceptible to this antimicrobial and one was resistant.

### Bacterial quantification

Bacterial quantification and isolation of specific bacterial species were performed on all samples. As such, 204 environmental samples were analyzed to evaluate the bacterial load on the BICU surfaces. In the isolation rooms, the cage surfaces were the most contaminated, with a mean bacterial quantification of 4.74 × 10^3^ ± 16.73 CFU/ml of swab suspension. In contrast, the examination tables revealed to be the surfaces with the lowest bacterial counts, presenting a mean of 2.90 × 10^2^ ± 33.04 CFU/ml of swab suspension. There was a statistically significant difference between the mean bacterial quantifications obtained for the different sampled surfaces from those rooms (*p* = 0.019). A higher bacterial quantification was observed in the dog isolation rooms when compared to the cat isolation rooms. However, this difference was not statistically significant (*p* = 0.073) (Table 2).

**Table 2:** Bacterial quantifications (CFU/ml of swab suspension) obtained from the different surfaces and rooms sampled

Rooms	Surfaces	Mean	SD	Median	Min.	Max.	***p***-value
		CFU/mL of swab suspension					
**ISOLATION ROOMS**							
C1^a^		9.51 × 10^2^	8.16	2.25 × 10^2^	0	8.15 × 10^3^	0.073
C2^a^		4.56 × 10^2^	13.61	1.50 × 10^2^	0	3.00 × 10^3^	
D1^b^		2.60 × 10^3^	14.03	4.25 × 10^2^	0	5.75 × 10^4^	
D2^b^		2.46 × 10^3^	22.14	1.00 × 10^3^	0	7.53 × 10^4^	
	Examination tables	2.90 × 10^2^	33.04	3.04 × 10^2^	0	5.76 × 10^3^	0.019^+^
	Cabinets	4.42 × 10^2^	6.09	1.75 × 10^2^	0	1.55 × 10^3^	
	Cabinets handles 2^c^	4.73 × 10^2^	9.83	1.75 × 10^2^	0	3.25 × 10^3^	
	Cabinets handles 3^d^	5.29 × 10^2^	17,73	2.00 × 10^2^	0	3.25 × 10^3^	
	Doorknobs	9.85 × 10^2^	16.68	7.33 × 10^2^	0	1.55 × 10^3^	
	Faucets	1.25 × 10^3^	3.81	4.00 × 10^2^	5.00 × 10^1^	8.15 × 10^3^	
	Cages	4.74 × 10^3^	16.73	1.10 × 10^3^	0	7.53 × 10^4^	
**STAFF AREA**							
Work room		3.96 × 10^3^	2.18	2.93 × 10^3^	1.00 × 10^2^	1.11 × 10^4^	
	Mouse	2,14 × 10^3^	3.15	1,33 × 10^3^	1.00 × 10^2^	7.00 × 10^3^	0.048^+^
	Keyboard	3.41 × 10^3^	1.19	2,60 × 10^3^	1.05 × 10^3^	6.95 × 10^3^	
	Telephone	6.33 × 10^3^	0.87	5,88 × 10^3^	2.00 × 10^3^	1.11 × 10^4^	
Preparatory room		6.98 × 10^5^	1.44 × 10^2^	3.60 × 10^4^	5.00 × 10^1^	1.00 × 10^9^	
	Food/Water bowls	9.86 × 10^3^	13.48	1.50 × 10^3^	5.00 × 10^1^	3.25 × 10^4^	< 0.01^+^
	Countertop	1.18 × 10^5^	7.37	3.19 × 10^4^	1.50 × 10^3^	5.55 × 10^5^	
	Hand-held sponge	2.09 × 10^7^	5.42	2.84 × 10^7^	9.45 × 10^6^	1.00 × 10^9^	

^a^ Cat isolation room

^b^ Dog isolation room

^c^ Pool of the last 2 cabinet handles

^d^ Pool of the first 3 cabinet handles

^+^ Statistically significant differences detected between groups

In the staff’s area, the analysis of variance revealed a statistically significant difference between the mean bacterial counts obtained on the different sampled surfaces, both in the work room (*p* = 0.048) and in the preparatory room (*p* < 0.01). In the work room, the most contaminated surface was the telephone, presenting a mean of 6.33 × 10^3^ ± 0.87 CFU/ml of swab suspension. In the preparatory room, it was the hand-held sponge, with a mean bacterial quantification of 2.09 × 10^7^ ± 5.42 CFU/ml of swab suspension. The comparison between the different rooms (work and preparatory rooms) also revealed a statistically significant difference (*p* < 0.01), with the preparatory being the most contaminated location (Table 2).

The new disinfection methods were applied to all surfaces and rooms of the isolation unit. However, only the surfaces previously identified as the most contaminated were selected for further analysis, namely the cages, the telephone, and the hand-held sponge from the isolation, work, and preparatory rooms, respectively.

**Fig. 1:**
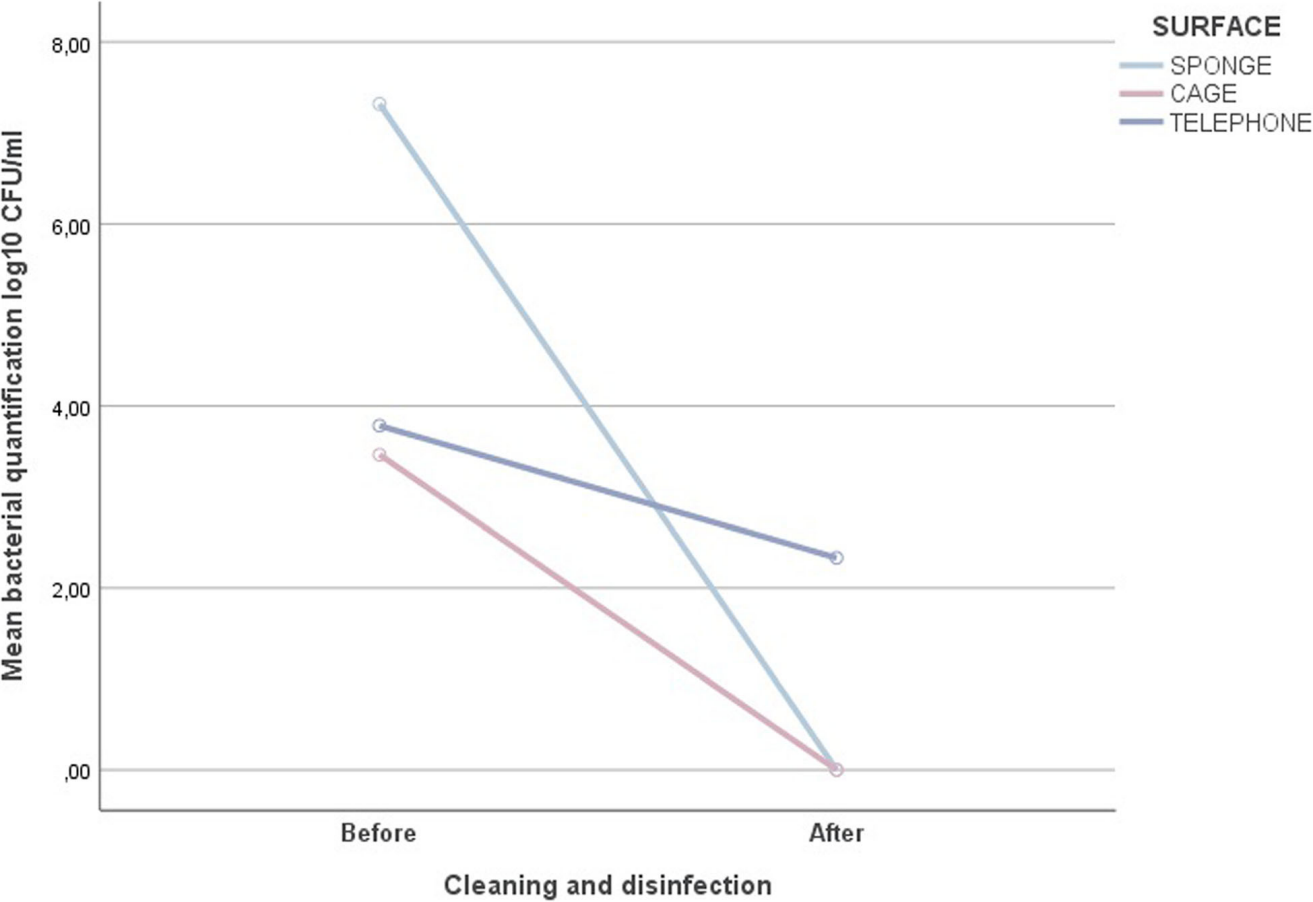
Logarithmic reduction of the bacterial quantification before and after the implementation of new disinfection protocols

After the implementation of the new disinfection protocols, bacterial load of the cage’s surfaces presented a log reduction of 3.68 log_10_; in the hand-held sponge, a logarithmic reduction of 7.32 log_10_ was observed; and the telephone surface presented a logarithmic reduction in its bacterial load of 1.41 log_10_ (Fig. 1). Statistical analysis using ANOVA test with repeated measures showed that the difference between the bacterial counts obtained before and after implementation of the new protocol on each surface was statistically significant (*p*-value < 0.05).

## Discussion

Hospital surfaces and medical equipment play an important role as reservoirs of nosocomial agents, allowing their dissemination [21]. For that reason, one of the main aims of this prospective study was to identify which potentially pathogenic microorganisms were present at BICU. Some bacterial species associated with nosocomial infections were isolated from selected surfaces, namely *Enterococcus* spp., *E. coli*, and *P. aeruginosa*. *S. aureus* was not recovered from any of the samples taken, even from human contact surfaces, where their presence would be expected. The prevalence of *S. aureus*, including methicillin-resistant *S. aureus* (MRSA) in veterinary hospital settings was already described by several authors [15, 17, 18, 22, 23]; Nevertheless, numerous factors may justify the different prevalence’s found between studies, including the present one, such as differences in study designs and methodology applied; the type of animal species admitted to the hospital; and the presence of colonized and/or infected patients or staff (which is the main reason associated with environmental contamination by this microorganism) [22]. The fact that different clinics/hospitals have different cleaning and disinfecting protocols is also relevant, as these differences may have a considerable impact in terms of environmental contamination [24].

Of the four microorganisms surveyed in this study, *Enterococcus* spp. were the most frequently recovered. With a prevalence of 11.3%, these bacterial agents were isolated from all surfaces sampled in the isolation rooms, having been mostly associated with cabinet handles and cages. Although preliminary and without statistical significance, these results are still relevant. Firstly, *Enterococcus* spp., being part of the animal’s intestinal microbiota, are usually eliminated through feces [25, 26]. Since the cages are constantly contaminated with animal excretions, the presence of these isolates in such areas is not surprising, as already described [19]. On another hand, its recovery from human contact surfaces should not have occurred. Due to the scope and design of this study, it was not possible to determine the source of these isolates, nor to determine whether there is an association between them. However, the fact that these bacteria were recovered from all surfaces of the isolation rooms, including those of exclusively human contact, suggests that clinician’s hands/gloves act as vehicles for a wide microbes dissemination, as already been reported by several authors [10, 27–29]. At BICU all employees who enter the isolation rooms must be properly equipped with personal protective equipment (PPE) and must comply with all established standard operating procedures (SOP). Thus, in this case, the use of protective barriers, such as gloves, may sometimes offer a false sense of protection to users, as advocated by other studies [30, 31], suggesting that their simple use is not enough to prevent the spread of microorganisms within the facilities.

*E. coli* was isolated from cages and the hand-held sponge, which was expected due to the frequent fecal contamination of these surfaces. However, the prevalence of this bacteria in BICU was relatively low (1.5%), compared to other studies [14, 32], revealing that the disinfection protocols established in BICU were effective at eliminating environmental contamination by Enterobacteriaceae.

In this work, the presence of *P. aeruginosa* was detected in 1.5% of the samples analyzed. The three positive isolates were obtained from swab samples taken from the feeding bowls and the hand-held sponge, located in the preparatory zone, and from a faucet located in an isolation room. This bacterial agent is known for colonizing water systems in hospitals [33–39], having been associated with the contamination of medical equipment and the hands of healthcare workers during washing procedures [34–37, 39]. Even so, although positive surfaces from this study are all associated with a water source, the possibility of occasional contamination from a patient colonized/infected by this bacterial agent cannot be ruled out, since no statistical relevance was found between its presence and the sampled surfaces. Out of the three positive isolates, the one recovered from the food/water bowls was resistant to imipenem. Being the carbapenems a class of antibiotics reserved for treating complicated infections caused by MDR bacteria, makes the emergence of resistance to these molecules of great concern (DGS 2017b). In this context, it would be interesting to carry out new laboratory tests on this isolate to confirm its resistance profile and evaluate its eventual carbapenemase enzyme production.

The frequent isolation of the same type of microorganisms from different hospital surfaces can be associated with two main situations: their constant reintroduction in these environments or failures in the cleaning and disinfection protocols applied, allowing the maintenance and survival of these agents for long periods in the environment [40, 41]. To conclude whether these surfaces are in fact reservoirs of the bacterial agents identified, it would be necessary to evaluate a larger sample size.

Regarding bacterial quantification, the present study showed that, in the BICU, it was the staff’s area that revealed the highest levels ​​of contamination, when compared to the isolation rooms. Although, to the authors’ knowledge, there are no other similar studies carried out in isolation units in the veterinary context, results can be explained by the fact that contact precautions and cleaning and disinfection protocols are most thoroughly performed at isolation rooms, since these are where infectious patients are hospitalized. Also, these controlled environments operate in a negative pressure system and with individualized HEPA air filters. Strict SOP are applied, including the mandatory use of PPE for anyone entering these rooms, and the disinfection of all surfaces which contacted, directly or indirectly, with patients is also obligatory [42].

Regarding the isolation rooms, the cages were the surfaces that showed a higher bacterial quantification. This result was expected since these large surfaces present the highest levels of dirt and contamination with all types of animal secretions, which represents a greater challenge in terms of cleaning and disinfection. In other studies carried out in veterinary hospitals, cages have also been identified as one of the most contaminated [17, 43].

At BICU, when a patient is examined, all surfaces and equipment used must be thoroughly cleaned and disinfected afterward. Therefore, the examination tables and the top of the cabinets, used for medical equipment storage, are the surfaces that are most frequently disinfected throughout the day. This fact may explain our results since these two surfaces had the lowest mean bacterial counts. On another end, the faucets, and handles, especially the doorknobs, presented significant bacterial counts. These surfaces are manipulated exclusively by humans and are considered by several authors as important reservoirs of various infectious agents in veterinary hospital environments [15, 18, 22, 40].

The preparatory room had the highest bacterial counts of all the analyzed rooms, possibly because it is a transition area between the isolation rooms and the rest of the facilities. This is where the dishes and other materials coming from the isolation rooms are washed and disinfected and where food preparation for patients is carried out; and, therefore, where the levels of organic matter and humidity are higher. In this room, the hand-held sponge was the most contaminated surface. This is an expected result given the porous nature of the object, which allows for easy retention of water and organic matter, thus gathering the ideal conditions for bacteria multiplication [44].

Of the 3 selected surfaces analyzed in the work room, the telephone had the highest bacterial quantification. This object had already been signalized by other studies as commonly contaminated by nosocomial agents [45]. Nevertheless, both the computer mouse and the keyboard also revealed considerable bacterial counts, probably because these devices are frequently used by several people throughout the day. Additionally, their cleaning and disinfection process is hampered by their very irregular and intricate structure [46, 47].

There are multiple possibilities regarding the approach to infection prevention and control programs in a hospital environment, with each institution being responsible for choosing and implementing measures that best adapt to its needs. Equally important is the development and mobilization of efforts to monitor the effectiveness of the actions taken, to enable the easy and quick optimization of established protocols (if so indicated), guaranteeing the best safety conditions for all who visit the facilities [13]. Despite the increasing relevance of nosocomial infections, the ideal cleaning and disinfecting method for controlling these infectious agents is still not known. Several authors have evaluated the effectiveness of different procedures and products in reducing the bacteria load of different hospital surfaces, having obtained, in general, very positive results [43, 45, 48–53]. Interestingly, although several protocols proved to be quite effective, in practice most institutions continue to reveal difficulties in this area, suggesting that probably other factors, such as the correct compliance to the planned measures, have a strong impact on the final results [29, 30, 48, 54].

With the implementation of the bacteriological control plan performed in this study, it was possible to identify flaws in the disinfection protocols previously applied in the BICU, namely the fact that a much higher disinfectant dilution was used compared to the product manufacturer’s recommendations. Those recommendations must always be followed to ensure the product’s optimal efficacy. As so, the concentration of the biocide was increased as recommended, and in addition, new time points were included in the environmental surfaces cleaning and disinfection daily schedule. These new disinfection strategies have been applied to all surfaces and rooms of the isolation unit, however, only the surfaces previously identified as the most contaminated were selected for further bacteriological analysis. Positive results were obtained, and statistically significant logarithmic reductions of 3.68 log_10_, 7.32 log_10_, and 1.41 log_10_ were observed for the cages, hand-held sponge, and telephone, respectively. Percentage wise, the total bacterial load reduction was approximately 100% for the cages and the hand-held sponge (99.9%) and 90 to 99% for the telephone. As such, this preliminary evaluation showed that the new disinfection strategies implemented at BICU seemed to be effective in reducing environmental contamination, including by some potentially nosocomial agents, although a wider sample size must be analyzed for definitive conclusions.

## Conclusions

This study showed that in BICU isolation rooms, cages and human contact surfaces had the highest bacterial contamination. Outside these rooms, the preparatory zone was the one that showed the most worrying results, in terms of total aerobic bacterial load and detection of a *P. aeruginosa* isolate resistant to imipenem in the feeding bowls. However, no other bacteria signaled in this environmental surveillance program were found, namely MRSA, VRE, and 3rd-generation cephalosporin-resistant *E. coli*. Results led to the immediate implementation of new disinfection protocols that reduced the total bacterial load and eliminated the target bacterial species from the analyzed surfaces.

With this study, it is possible to observe that bacteriological surveillance programs in hospital environments appear to be useful tools for monitoring surface contamination levels and, eventually, identifying potential sources of infection and/or breaches in the established disinfection protocols. In this way, it is possible to easily and quickly implement corrective actions that may be fundamental to preventing future infections. Even so, the levels of bacterial contamination at BICU were consistently lower than those observed in other studies carried out in several departments of veterinary hospitals, other than isolation. These results, although preliminary, may contribute to highlight the importance of infection prevention and control measures, as fundamental for reducing the spread of infectious agents in the hospital environment and, consequently, for increasing the quality and safety of the service provided. Continuous development of scientific research on this subject is urgent, especially in veterinary medicine, where the available data on nosocomial infections are still very scarce.

## Methods

### Description of the isolation unit

The Teaching Hospital (TH) of the Faculty of Veterinary Medicine of the University of Lisbon (FMV-ULisbon) includes an isolation unit for the hospitalization of dogs and cats with confirmation (or clinical suspicion awaiting diagnosis) of infectious diseases [42]. The Biological Isolation and Containment Unit (BICU) is located in a separate building from the rest of the TH, and, in these facilities, each isolation room works with negative differential pressure, HEPA air filters, video surveillance system, and specific SOP. These include contact precautions, such as the use of PPE, promotion of hand hygiene, and application of rigorous disinfection protocols of medical equipment and environmental surfaces [42].

The BICU comprises four isolation rooms, two for dogs and two for cats, all with the same type of equipment and layout; a work room with a computer for internet access and patient clinical records; a preparatory area for washing and disinfecting materials coming from the isolation rooms, and with access to the unit’s storeroom; and an anteroom used for the entry and exit of animals from the facilities and follow-up and/or reference appointments.

### Sampling procedures

For this study, swab samples were collected from several surfaces in different areas of the BICU (isolation, work, and preparatory rooms) in a total of 426 environmental samples (204 for bacterial identification, 204 for bacterial quantification, and 18 for evaluating the effectiveness of the new disinfecting protocol) (Table 3). Surfaces were selected for sampling after a careful observation of the procedures performed at the different areas of BICU for a week, allowing to select the ones more exposed to bacteria contamination and cross-dissemination.

**Table 3:** Description of the surfaces sampled in different areas of the BICU for microbiological analysis

Sampling areas	Surfaces	Number of samples (n=)
**ISOLATION ROOMS**		
C1^a^		87
C2^a^		87
D1^b^		87
D2^b^		87
	Doorknobs	48
	Faucets	48
	Cabinets	48
	Cabinet handles ^c^	96
	Examination tables	48
	Cages	60
**STAFF AREA**		
Work room		39
	Keyboard	12
	Mouse	12
	Telephone	15
Preparatory		39
	Food/water bowls ^d^	12
	Countertop	12
	Hand-held sponge	15

^a^ Cat isolation room

^b^ Dog isolation room

^c^ Divided into two separate samples, one representing a pool of the first three handles and other a pool of the last two

^d^ Samples taken as a pool of 3 bowls

Sampling days were chosen randomly, with at least two-week intervals, in a total of 6 sampling days per room for tasks 1 and 2 (bacterial quantification and identification) and of 3 sampling days per room for task 3 (evaluation of the new disinfecting protocols). Both sampling days and hours were scheduled randomly in order to create unpredictability and thus eliminate potential bias.

Samples were collected using disposable cotton-tip swabs soaked in a sterile saline solution (NaCl 0.9%). For larger surfaces, such as the cabinets, examination tables, cages, and countertop, an area of ​​10x10cm was delimited with the aid of a sterile tool. Swabs were placed in 1 ml of sterile saline solution and taken immediately for bacteriological analysis at the Laboratory of Microbiology and Immunology of FMV-ULisbon.

### Determination of the bacterial load of selected surfaces

For determining the bacterial load of selected surfaces, a total of 204 samples were analyzed. Swab sample suspensions in saline solution were homogenized and serial diluted from 10^− 1^ to 10^− 3^, apart from those originating from the hand-held sponge. This object is used to remove the organic matter and other debris from the food/water bowls before its disinfection process. As so, due to the expected high bacterial load, those suspensions were diluted up to 10^− 7^. From each dilution, including the original suspension, 100 μL were inoculated on a nonspecific enrichment culture medium, Brain Heart Infusion agar (BHI – Oxoid® ref. CM1136) and incubated at 37 °C for 48 h. Bacterial quantification was performed by determining the colony-forming units per milliliter of swab suspension (CFU/ml swab suspension) at 24 h and 48 h.

### Isolation of bacterial species associated with nosocomial infections

A total of 204 BICU surface samples were analyzed for the presence of nosocomial species. Swab sample suspensions in saline solution were homogenized and serial diluted from 10^− 1^ to 10^− 2^, apart from those originating from the hand-held sponge which were diluted up to 10^− 6^. Next, 100 μL from each suspension, including the original suspension and the dilutions, were inoculated on Slanetz & Bartley agar (S&B - Scharlau® ref.01–579-500), MacConkey agar (MCK- Oxoid® ref.CM0115) and Mannitol Salt agar (MSA- Scharlau® ref.01–116-500) and incubated at 37 °C for 48 h. After incubation, the colonies with macroscopic characteristics compatible with the target species were quantified and inoculated on Columbia agar medium (COS - Biomérieux® ref. 43,041– Blood Agar culture media + 5% sheep blood) to be further characterized. Isolates that originated yellow colonies on MSA, characterized as Gram-positive cocci, catalase-positive, mannitol fermenters, and coagulase-positive were presumptively identified as *S. aureus*. Pink colonies on S&B agar, characterized as Gram-positive cocci, catalase-negative, and with aesculin hydrolysis on Edward’s agar were presumptively identified as *Enterococcus* spp*.* Large pale lactose negative colonies on MCK agar, characterized as Gram-negative bacilli and oxidase-positive were presumptively identified as *P. aeruginosa*. Finally, pink lactose positive colonies on MCK agar, characterized as Gram-negative bacilli, oxidase-negative, and indole and motility positive, Voges-Proskauer and citrate negative on IMViC test, were presumptively identified as *E. coli*.

The susceptibility profile of the isolates presumptively identified as belonging to the target species to selected antimicrobials was characterized using the Kirby-Bauer disc diffusion method, following Clinical and Laboratory Standards Institute protocols [20]. Thus, enterococci susceptibility to vancomycin (30 μg) and *P. aeruginosa* isolates susceptibility to imipenem (10 μg) were determined, while the phenotypic production of ESBL by the *E. coli* isolates was evaluated using cefotaxime (30 μg), amoxicillin + clavulanic acid (20/10 μg), and ceftazidime (30 μg). All antibiotic disks were purchased from Oxoid®.

### Implementation of new surface disinfection protocols

BICU surface disinfection protocols were optimized and their application to the surfaces with higher bacterial counts (one from each sampling area of the BICU – isolation, work, and preparatory rooms) was evaluated.

Before this study, surface disinfection was performed by using a very diluted (unknown) concentration of the disinfectant Virkon®S applied to all surfaces after direct or indirect contact with patients. The new disinfection protocols considered 2 key points: an increase in the frequency of surface disinfection procedures, carried out every morning before the daily work routine started, and after every usage or contact with patients; and an increase in the concentration and frequency of preparation of the disinfectant used on the facilities (Virkon® S). The disinfectant solution was made every week at a dilution of 1:100 since according to the manufacturer’s recommendations a 1% solution is expected to have an effective biocidal action during 5–7 days [55].

After the implementation of the new protocols, only the most contaminated surface of each room was selected to be reevaluated, namely the cages, hand-held sponge, and the telephone; even so, every surface and room of the BICU has undergone changes in their disinfecting protocols. At this stage, samples were taken according to the same protocol as previously described. Samples were taken 15 days apart, making a total of 3 sampling days per room.

### Data analysis

Statistical analysis was performed using the IBM SPSS® Statistics 26 program.

For statistical purposes, microbial quantifications were converted to base 10 logarithms, using the formula log_10_ (CFU/mL + 1), allowing to include all null quantifications in the analysis.

The analysis of variance of the bacterial load of selected surfaces was performed using one-way ANOVA, while the variance between results from the different disinfection protocols was evaluated using ANOVA with repeated measures. Fisher’s exact test was performed to evaluate the association between the presence of the different microorganisms identified and the environmental surfaces. Statistical significance was determined at the cut-off value of 0,05 (*p* < 0,05).

## Data Availability

The datasets used and/or analyzed during the current study are available from the corresponding author on reasonable request.

## Abbreviations

NI: Nosocomial Infections
TH: Teaching Hospital
FMV-ULisbon: Faculty of Veterinary Medicine of University of Lisbon
BICU: Biological and Isolation Containment Unit
SOP: Standard Operating Procedures
PPE: Personal Protective Equipment
HEPA: High Efficiency Particulate Air
MDR: Multidrug-resistant
CLSI: Clinical & Laboratory Standards Institute
BHI: Brain Heart Infusion
MSA: Mannitol Salt Agar
S&B: Slanetz & Bartley
MCK: MacConkey
MRSA: Methicillin-resistant *S. aureus*
VRE: Vancomycin- resistant *Enterococcus*
ESBL: Extended-spectrum beta-lactamase
CFU: Colony Forming Units
